# Neutrophil‐to‐Lymphocyte Ratio as an Independent Predictor of Adverse Short‐Term Functional Outcomes After Reperfusion Therapy in Acute Ischemic Stroke

**DOI:** 10.1002/brb3.71122

**Published:** 2025-12-10

**Authors:** Jun‐ying Li, Jun Li, Zhong‐jiao Lu, Wen Zhou, Yan‐hui Li, Yong‐jiang Luo, Xue‐min Zhong, Jian Wang, Jing Gou, Lan‐ying He

**Affiliations:** ^1^ Department of Neurology West China School of Medicine, Sichuan University, Sichuan University affiliated Chengdu Second People's Hospital, Chengdu Second People's Hospital Chengdu P. R. China; ^2^ Bai Lian chi Community Health Center, Chenghua District Chengdu P. R. China; ^3^ Department of Neurology, Renji Hospital Shanghai Jiao Tong University School of Medicine Shanghai P. R. China

**Keywords:** acute ischemic stroke, neutrophil‐to‐lymphocyte ratio (NLR), reperfusion therapy, short‐term outcomes

## Abstract

**Background and purpose:**

Intravenous thrombolysis (IVT) and endovascular thrombectomy (EVT) are primary treatments for acute ischemic stroke (AIS), but their efficacy is limited. This study aims to evaluate the short‐term predictive value of the neutrophil‐to‐lymphocyte ratio (NLR) in AIS patients undergoing reperfusion therapies.

**Method:**

AIS patients who underwent IVT and/or EVT at Chengdu Second People's Hospital were continuously enrolled from January 2020 to September 2024. NLR was calculated from blood samples taken before treatment. Primary outcomes were functional status at discharge (assessed using the modified Rankin Scale [mRS]), while secondary outcomes included in‐hospital mortality and any intracranial hemorrhage (ICH). Statistical analyses included logistic regression and receiver operating characteristic (ROC) curve analysis.

**Results:**

Among 817 patients, 327 (40.0%) exhibited poor functional outcomes at discharge. NLR positively correlated with the National Institutes of Health Stroke Scale score (*ρ* = 0.298, *p* < 0.001). Univariate analysis showed a significant association between NLR and poor functional outcomes at discharge, higher in‐hospital mortality, and increased ICH incidence. After adjusting for confounders, NLR remained an independent predictor of functional outcomes (odds ratio 1.092; 95% confidence interval [CI] 1.006–1.185; *p* = 0.036). ROC analysis showed that NLR could predict functional outcomes with a cutoff value of 3.66 and an area under the curve of 0.679 (95% CI 0.641–0.717, *p* < 0.001).

**Conclusions:**

NLR is an independent predictor of short‐term functional outcomes and complications in AIS patients receiving reperfusion therapies, serving as a valuable tool for early prognosis and clinical decision‐making.

## Graphical abstract

Elevated neutrophil‐to‐lymphocyte ratio (NLR) independently predicts unfavorable short‐term functional outcomes in acute ischemic stroke patients receiving reperfusion therapy, highlighting its potential as a simple, accessible biomarker for early risk stratification and prognosis evaluation.

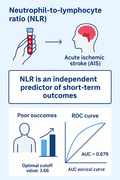

## 1 Introduction

Intravenous thrombolysis (IVT) and endovascular thrombectomy (EVT) remain the cornerstone therapies for acute ischemic stroke (AIS). Nevertheless, their overall effectiveness is suboptimal, with only about one‐third to one‐half of patients achieving favorable long‐term functional recovery (Semerano et al. 2019; The National Institute of Neurological Disorders and Stroke rt‐PA Stroke Study Group 1995). Clinical practice data have shown that, even after technically successful reperfusion, a substantial proportion of patients experience futile recanalization and unsatisfactory outcomes (Baek and Kim 2019; Goyal et al. 2016). A major factor contributing to this discrepancy is cerebral ischemia‐reperfusion injury, a process characterized by oxidative stress, free radical release, and subsequent neuronal damage (X. Li et al. 2022).

Inflammation plays a central role in the pathophysiology of AIS, aggravating tissue injury and worsening prognosis (Iadecola and Anrather 2011; Kim et al. 2016). In particular, infiltration of circulating leukocytes—most notably neutrophils—has been identified as a critical driver of secondary injury (Ishikawa et al. 2004). The neutrophil‐to‐lymphocyte ratio (NLR), calculated from routine peripheral blood counts, has recently attracted attention as a convenient marker of systemic inflammation and a potential prognostic factor in AIS (Ethier et al. 2017; M. X. Li et al. 2014). During the acute phase, neutrophils accumulate in ischemic tissue, releasing cytokines, chemokines, and matrix metalloproteinase‐9 (MMP‐9), which disrupt the blood‐brain barrier (BBB), enlarge infarct volume, predispose to hemorrhagic transformation (HT), and worsen neurological function (Kim et al. 2016; Otxoa‐de‐Amezaga et al. 2019). Conversely, lymphocytes are thought to limit inflammation and promote tissue repair, thereby contributing to functional recovery (S. Li et al. 2021). Additionally, neutrophil‐released inflammatory factors, such as cytokines, chemokines, and MMP‐9, induce oxidative stress, platelet accumulation, and BBB damage, further accelerating brain injury (Jickling et al. 2015). Because NLR reflects the balance between pro‐inflammatory neutrophil activity and immunoregulatory lymphocyte function, it has been proposed as a surrogate indicator of the host inflammatory response (Song et al. 2019; Walsh, Cook, Goulder, Justin, and Keeling 2005). Recent studies suggest that post‐stroke immune alterations, including elevated NLR, may influence the neurovascular unit, contribute to reperfusion‐related injury, and increase the risk of symptomatic intracranial hemorrhage (sICH) (Sharma et al. 2021). Several investigations have reported that patients with higher admission NLR levels are more likely to develop sICH and to have unfavorable 3‐month outcomes following IVT or EVT (Maestrini et al. 2015; Semerano et al. 2019).

Although several previous studies have assessed functional status at discharge following reperfusion therapy, few have stratified patients according to their discharge status to independently analyze the associated influencing factors and evaluate the predictive value of NLR for this outcome. To fill this gap, the present study investigates the predictive utility of leukocyte parameters—particularly neutrophil count and NLR—for short‐term outcomes in AIS patients receiving reperfusion therapy.

## 2 Materials and Methods

### 2.1 Subjects

This retrospective, single‐center analysis enrolled consecutive AIS patients aged 18 years or older who underwent IVT and/or EVT at Chengdu Second People's Hospital between January 2020 and September 2024. Patients were excluded if they met any of the following criteria: (1) a pre‐stroke modified Rankin Scale (mRS) score > 2; (2) **patients who had experienced an acute pulmonary infection or urinary tract infection within 2 weeks before admission or had a recent infection (within the past 2 weeks) requiring anti‐infective therapy;** (3) a history of chronic inflammatory disease or ongoing corticosteroid therapy; or (4) incomplete baseline data, particularly missing neutrophil or lymphocyte counts. To extend treatment beyond the conventional time window, patient selection for reperfusion was supported by multimodal MRI or CT perfusion imaging. IVT was administered within 6 h of symptom onset or within 4.5 h of last known well, whereas EVT was carried out within 6 h after onset (Powers et al. 2019). Decisions regarding anesthesia type and interventional technique were made at the discretion of the treating neurointerventionalists. Clinical reassessment included a follow‐up CT scan and National Institutes of Health Stroke Scale (NIHSS) evaluation at approximately 24 h post‐treatment to detect intracranial hemorrhage (ICH). The study protocol was approved by the Ethics Committee of Chengdu Second People's Hospital, and written informed consent was obtained from each participant or a legally authorized representative. All procedures adhered to the ethical principles of the Declaration of Helsinki.

### 2.2 Data Collection

Clinical information was systematically collected, including demographic variables (age and sex) and vascular risk factors such as hypertension, diabetes mellitus, hyperlipidemia, coronary heart disease, atrial fibrillation, transient ischemic attack, peripheral arterial disease, and history of ischemic stroke. Lifestyle habits, including tobacco and alcohol use, were also recorded. On admission, routine laboratory investigations and blood pressure measurements were performed. Neurological status was assessed with the NIHSS and functional disability was rated by the mRS at baseline and at discharge. Details of reperfusion therapy, including treatment type and onset‐to‐reperfusion time, were documented. Stroke subtype was determined using the modified Trial of ORG 10172 in Acute Stroke Treatment (TOAST) classification system. Peripheral venous blood was drawn from the unaffected arm while the patient was in the supine position, prior to reperfusion and within 24 h of symptom onset. The NLR was calculated as the ratio of absolute neutrophil count to lymphocyte count. We assessed delayed reperfusion in patients undergoing EVT by evaluating their reperfusion status. Recanalization status was determined by a neuro‐interventionist with over 10 years of experience using the modified Thrombolysis in Cerebral Infarction (mTICI) scale based on post‐procedural digital subtraction angiography. Successful recanalization was defined as mTICI grade 2b or 3.

### 2.3 Clinical Outcome

Stroke severity was determined using the NIHSS (Adams et al. 1999), with scores of ≥ 8 categorized as moderate‐to‐severe events (Hess et al. 2017). The primary outcome was functional status at discharge, evaluated by the mRS; favorable recovery was defined as mRS ≤ 2, while unfavorable outcome was defined as mRS > 2 (Hill et al. 2020). Secondary endpoints included the development of ICH of any type and in‐hospital all‐cause mortality.

### 2.4 Statistical Analysis

Statistical analyses were conducted using SPSS version 23.0 (SPSS Inc., Chicago, IL, USA) and GraphPad Prism 9.5.1 (GraphPad Software, San Diego, CA, USA). Categorical variables were summarized as counts and percentages and compared with the chi‐square test. The distribution of continuous variables was examined using the Kolmogorov–Smirnov test; data with skewed distribution were reported as medians with interquartile ranges (IQRs) and compared using the Mann–Whitney U test. Associations between continuous variables were explored with Spearman's rank correlation. To identify independent predictors of outcomes, multivariate logistic regression was performed, adjusting for potential confounders, and results were expressed as odds ratios (OR) with 95% confidence intervals (CIs). The discriminatory power of NLR for functional prognosis was evaluated by receiver operating characteristic (ROC) curve analysis, and the optimal cutoff point was defined according to the Youden index. Additionally, a generalized linear model with binary logistic regression was constructed by incorporating multiple covariates to generate predicted probabilities, with model performance further assessed through ROC analysis. A two‐tailed *p* value ＜ 0.05 was considered statistically significant.

## 3 Results

### 3.1 Baseline Characteristics

**FIGURE 1:**
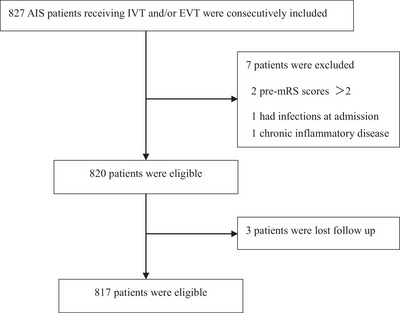
Study flow chart. AIS, acute ischemic stroke; EVT, endovascular thrombectomy; IVT, intravenous thrombolysis; mRS, modified Rankin Scale.

A total of 827 AIS patients who received reperfusion therapy were screened, and 817 met the eligibility criteria and were included in the final analysis (Figure 1). Baseline demographics and clinical features are presented in Table 1. The mean age was 70.32 ± 13.20 years, and 472 patients (57.8%) were male. The median NIHSS score at admission was 7 (IQR 3–14). IVT was administered to 68.0% of patients, EVT to 18.4%, and both treatment to 13.6%. The median onset‐to‐reperfusion time was 150 min (IQR 90–238, 75 min). Based on TOAST criteria, 45.1% were classified as large‐artery atherosclerosis, 31.8% as cardioembolism, and 23.1% as other causes. During hospitalization, 84 patients (10.3%) developed ICH, and 51 (6.24%) died. At discharge, 490 (59.98%) achieved a good functional outcome (mRS ≤ 2). Compared with those with good outcomes, patients with poor outcomes were older (*p* ＜ 0.001), fewer male (*p* = 0.014), and had lower rates of smoking (*p* = 0.001), alcohol use (*p* = 0.010), but had higher rates of diabetes (*p* = 0.024), and atrial fibrillation (*p* ＜ 0.001). They also exhibited higher leukocyte and neutrophil counts, and lower lymphocyte counts, as well as increased NLR and urea nitrogen levels, but lower uric acid and uric acid‐to‐creatinine ratios (all *p* ≤ 0.001). In addition, they had higher admission NIHSS and mRS scores (*p*＜0.001), longer onset‐to‐reperfusion time (*p* = 0.004), and a greater incidence of ICH (*p* ＜ 0.001). Significant differences were also observed in reperfusion type and etiological subtype (both *p* ＜ 0.001).

**TABLE 1:** Baseline characteristics of the study population included in the study.

	Total (*N* = 817)	Good outcome (*N* = 490)	Poor outcome (*N* = 327)	*p*
Age (years, mean ± SD)	70.32 ± 13.20	68.39 ± 13.03	73.23 ± 12.95	˂ 0.001^*^
Male, *N* (%)	472 (57.8)	300 (61.2)	172 (52.6)	0.014^*^
Smoking, *N* (%)	269 (32.9)	183 (37.3)	86 (26.3)	0.001^*^
Drinking, *N* (%)	204 (25.0)	138 (28.2)	66 (20.2)	0.010^*^
Hypertension, *N* (%)	464 (56.8)	277 (56.5)	187 (57.2)	0.853
Diabetes, *N* (%)	208 (25.4)	111 (22.7)	97 (29.7)	0.024^*^
Hyperlipidemia, *N* (%)	15 (1.8)	11 (2.2)	4 (1.2)	0.287
Coronary artery disease, *N* (%)	63 (7.7)	33 (6.7)	30 (9.2)	0.200
Arterial fibrillation, *N* (%)	140 (17.1)	63 (12.9)	77 (23.5)	˂ 0.001^*^
History of stroke, *N* (%)	115 (14.1)	66 (13.5)	49 (15.0)	0.542
History of TIA, *N* (%)	4 (0.5)	3 (0.6)	1 (0.3)	0.539
History of PAD, *N* (%)	8 (1.0)	6 (1.2)	2 (0.6)	0.383
Systolic blood pressure (mmHg, mean ± SD)	147.33 ± 22.91	146.97 ± 22.24	147.87 ±23.90	0.581
Diastolic blood pressure (mmHg, mean ± SD)	82.44 ± 15.20	82.67 ± 14.52	82.10 ± 16.19	0.578
White blood cell count (×10^9^/L), median (IQR)	7.63 (6.12–9.50)	7.10 (5.90–8.63)	8.57 (6.92‐10.66)	˂ 0.001^*^
Neutrophil count (×10^9^/L), median (IQR)	4.95 (3.71–6.90)	4.4 (3.4–6.0)	6.0 (4.5–8.3)	˂ 0.001^*^
Lymphocyte count (×10^9^/L), median (IQR)	1.50 (1.03–2.10)	1.6 (1.2–2.3)	1.30 (0.83–1.85)	˂ 0.001^*^
Neutrophil‐to‐lymphocyte ratio (NLR), median (IQR)	3.18 (1.96–5.85)	2.62 (1.75–4.34)	4.53 (2.48–8.79)	˂ 0.001^*^
Blood platelet count (×10^9^/L), median (IQR)	180 (144–226)	179.5 (144–224)	181 (145–232)	0.528
Uric acid (µmol/L), median (IQR)	331.0 (269.0–403.5)	342.0 (282.75–405.3)	314.0 (237.0–402.0)	0.001*
Creatinine (µmol/L), median (IQR)	74.0 (62.0–90.0)	74.0 (62.0–90.0)	74.0 (62.0–91.0)	0.898
Urea nitrogen (mmol/L), median (IQR)	5.7 (4.6–7.2)	5.5 (4.6–6.8)	6.1 (4.7–7.8)	˂ 0.001*
Uric acid/creatinine, median (IQR)	4.42 (3.47–5.42)	4.7 (3.8–5.5)	4.1 (3.2–5.3)	˂ 0.001*
Baseline NIHSS score, median (IQR)	7 (3–14)	4 (2–8)	14 (9–18)	˂ 0.001^*^
Baseline mRS score, median (IQR)	4 (3–4)	3 (2–4)	4 (4–5)	˂ 0.001^*^
Reperfusion type				˂ 0.001^*^
Intravenous thrombolysis, *N* (%)	556 (68.0)	411 (83.9)	145 (44.4)	
Endovascular treatment, *N* (%)	150 (18.4)	42 (8.6)	108 (33.0)	
Both, *N* (%)	111 (13.6)	37 (7.5)	74 (22.6)	
Onset‐to‐reperfusion time, median (IQR), min	150 (90–238.75)	138.5 (87.75–209.25)	168.5 (100–311.25)	0.004^*^
Successful recanalization (mTICI 2b or 3), *N* (%)	6 (2.30%)	0 (0%)	6 (3.30%)	0.182
Etiological classification				˂ 0.001^*^
Large artery atherosclerosis, *N* (%)	368 (45.1)	218 (44.5)	150 (45.9)	
Cardio embolism, *N* (%)	260 (31.8)	117 (23.9)	143 (43.7)	
Lacunar, *N* (%)	160 (19.6)	132 (26.9)	28 (8.6)	
Undetermined, *N* (%)	20 (2.4)	18 (3.7)	2 (0.6)	
Other known causes, *N* (%)	9 (1.1)	5 (1.0)	4 (1.2)	
ICH, *N* (%)	84 (10.3)	21 (4.3)	63 (19.3)	˂ 0.001^*^

*Note*: Values are presented as medians (interquartile range, IQR) for continuous variables and numbers (percentages) for categorical variables. Plus–minus values are means ± SD.

Abbreviations: ICH, intracranial hemorrhage; IQR, interquartile range; mRS, the modified Rankin scale; mTICI, the modified Thrombolysis in Cerebral Infarction; NIHSS, Scores on the National Institutes of Health Stroke Scale, PAD, peripheral arterial disease, TIA, transient ischemic attacks.

^*^ *p* < 0.05

We compared the NLR values among the IVT, EVT, and IVT + EVT groups and found a significant trend of IVT < EVT < IVT + EVT (Table S1). Although this suggests that the inflammatory response may be higher in the EVT and IVT + EVT groups than that in the IVT group, we further performed stratified analyses to assess the impact of NLR on functional outcomes within each subgroup. Specifically, NLR was compared between patients with good (mRS ≤ 2) and poor (mRS > 2) outcomes at discharge. Across all three treatment modalities, patients with poor outcomes showed significantly higher NLR values (*p* < 0.001 for IVT, *p* = 0.040 for EVT, and *p* = 0.023 for IVT + EVT; Table S2). Moreover, to control for potential confounders, a binary logistic regression analysis including reperfusion therapy type as a covariate was conducted. The results showed that the treatment modality itself was not significantly associated with discharge outcomes (*p* > 0.05, Table 3). In multivariate logistic regression, independent predictors of poor functional outcome at discharge included higher NLR (*p* = 0.036), admission NIHSS score (*p* ＜ 0.001), baseline mRS score (*p* ＜ 0.001), onset‐to‐reperfusion time (*p* = 0.019), and presence of ICH (*p* ＜ 0.001) (Table 3).

### 3.2 Correlation Between NLR and Stroke Severity

**FIGURE 2:**
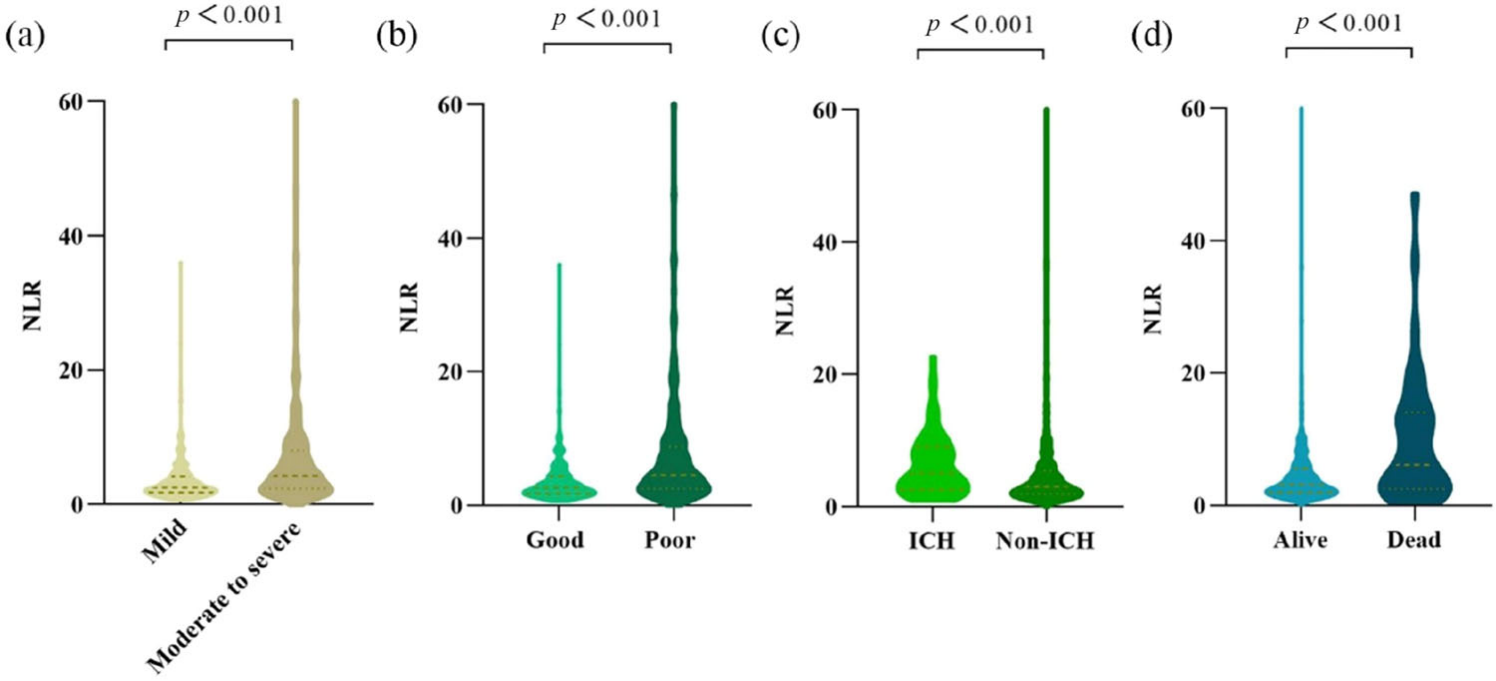
Comparison of NLR between patients between different groups. (a) Comparison of NLR between patients stratified by stroke severity; (b–d) comparison of NLR according to mRS score at discharge, presence and absence of ICH, and survival status (alive or dead) in hospital. mRS, modified Rankin Scale; NLR, neutrophil‐to‐lymphocyte ratio.

NLR was positively correlated with admission NIHSS scores (*ρ* = 0.298, *p* < 0.001). Moreover, patients presenting with moderate‐to‐severe stroke exhibited significantly higher NLR values than those with mild stroke (4.28 [2.38–8.0] vs. 2.50 [1.76–4.19]; *p* < 0.001; Figure 2a).

### 3.3 Association of Higher NLR With Poor Functional Outcome

In the univariate analysis, patients with unfavorable functional outcomes had significantly higher NLR values compared with those who achieved good recovery (4.53 [2.48–8.79] vs. 2.62 [1.75–4.34]; *p* < 0.001; Table 2; Figure 2b). After adjusting for confounders, multivariate logistic regression demonstrated that elevated NLR remained an independent predictor of poor outcome (OR 1.092; 95% CI 1.006–1.185; *p* = 0.036; Table 3). Additional independent predictors included admission NIHSS score, baseline mRS scores, and onset‐to‐reperfusion time (Table 3).

**TABLE 2:** Comparison of laboratory data according to different clinical outcome at discharge.

	Good outcome (*N* = 490)	Poor outcome (*N* = 327)	*p*	Alive (*N* = 766)	Dead (*N* = 51)	*p*	Non‐ICH (*N* = 733)	ICH (*N* = 84)	*p*
Neutrophil count (×10^9^/L), median (IQR)	4.40 (3.40–6.00)	6.00 (4.50–8.30)	˂ 0.001^*^	4.84 (3.70–6.76)	6.60 (4.90–10.20)	˂ 0.001^*^	4.81 (3.70–6.70)	6.35 (4.26–8.48)	˂ 0.001^*^
Lymphocyte count (×10^9^/L), median (IQR)	1.60 (1.20–2.30)	1.30 (0.83–1.85)	˂ 0.001^*^	1.50 (1.10–2.10)	1.0 (0.7–1.9)	0.002^*^	1.50 (1.10–2.10)	1.17 (0.80–1.60)	˂ 0.001^*^
NLR, median (IQR)	2.62 (1.75–4.34)	4.53 (2.48–8.79)	˂ 0.001^*^	3.10 (1.95–5.51)	6.13 (2.48–14.0)	˂ 0.001^*^	3.00 (1.90–5.45)	5.08 (2.53–9.80)	˂ 0.001^*^

Abbreviations: ICH, intracranial hemorrhage; IQR, interquartile range; NLR, neutrophil‐to‐lymphocyte ratio.

*p < 0.05.

**TABLE 3:** Multivariable logistic regression results of NLR in predicting poor functional outcome at discharge.

Variable	*β*	SE	Adjusted OR	95% CI	*p*
NLR	0.088	0.042	1.092	1.006–1.185	0.036*
Baseline NIHSS	0.138	0.026	1.148	1.090–1.209	˂ 0.001*
Baseline mRS	0.535	0.150	1.708	1.274–2.290	˂ 0.001*
Onset‐to‐reperfusion time	0.001	0.001	1.001	1.000–1.003	0.019*
Gender	0.128	0.256	1.137	0.688–1.878	0.616
Age	0.015	0.009	1.016	0.998–1.034	0.084
Drinking	−0.007	0.322	0.993	0.528–1.865	0.982
Smoking	−0.003	0.324	0.997	0.528–1.882	0.993
Diabetes	−0.381	0.223	0.683	0.441–1.057	0.087
Arterial fibrillation	−0.043	0.297	0.958	0.535–1.714	0.884
White blood cell count	−0.088	0.076	0.916	0.789–1.064	0.250
Neutrophil count	0.138	0.099	1.148	0.944–1.394	0.166
Lymphocyte count	0.204	0.131	1.227	0.949–1.585	0.118
Uric acid	−0.001	0.001	0.999	0.997–1.001	0.447
Urea nitrogen	−0.001	0.008	0.999	0.983–1.014	0.873
Uric acid/creatine	−0.009	0.079	0.991	0.849–1.157	0.909
Reperfusion type					0.463
	0.354	0.385	1.425	0.670–3.032	0.358
	−0.066	0.308	0.937	0.513–1.711	0.831
Etiological classification					0.108
	−1.625	0.976	0.197	0.029–1.335	0.096
	0.299	0.272	1.348	0.791–2.297	0.272
	1.062	0.829	2.893	0.569–14.700	0.200
	0.624	0.359	1.866	0.923–3.773	0.082
ICH	−1.375	0.338	0.253	0.130–0.491	˂ 0.001*

Abbreviations: 95%CI, 95% confidence intervals; ICH, intracranial hemorrhage; mRS, the modified Rankin scale; NIHSS, Scores on the National Institutes of Health Stroke Scale; NLR, neutrophil‐to‐lymphocyte ratio; OR, odds ratios. *p＜0.05.

### 3.4 Association of Higher NLR With In‐Hospital Mortality and ICH

**FIGURE 3:**
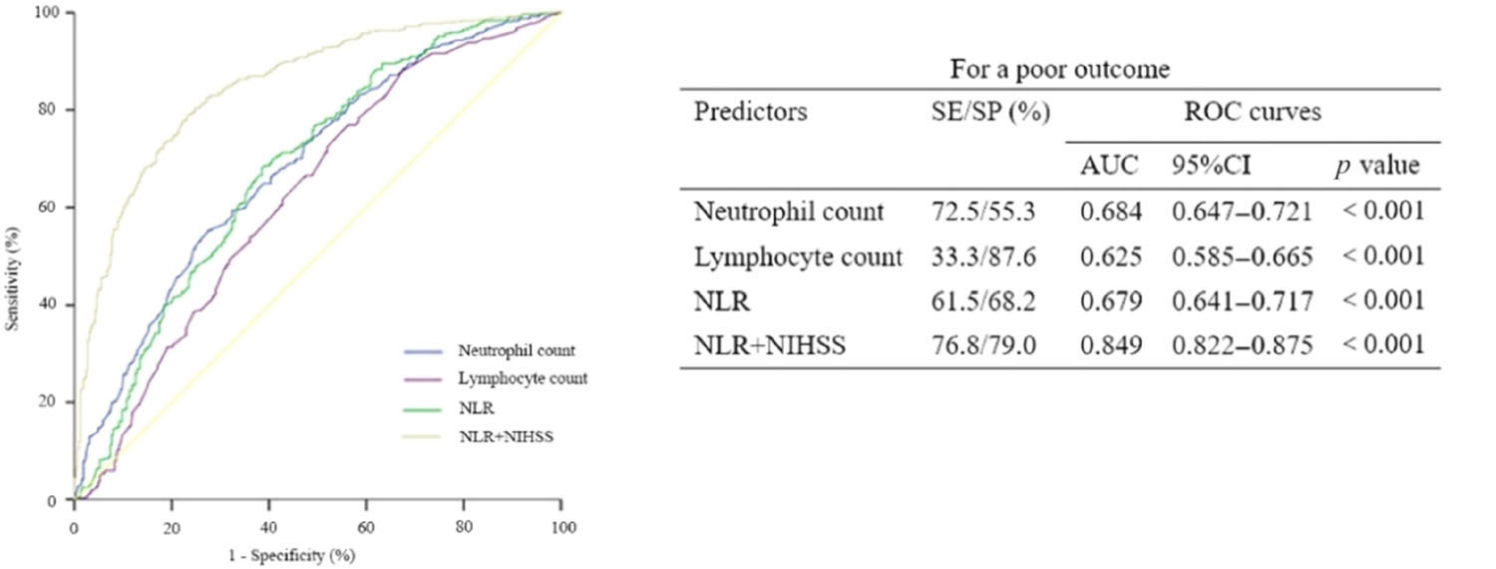
Prognostic accuracy of the NLR as a predictor of functional outcome at discharge after stroke. 95%CI, 95% confidence interval; AUC, area under the curve; NIHSS, Scores on the National Institutes of Health Stroke Scale; NLR, neutrophil‐to‐lymphocyte ratio; ROC, receiver operating characteristic; SE, sensitivity; SP, specificity.

In this cohort, 51 patients (6.24%) died during hospitalization, whereas 766 (93.76%) survived. ICH occurred in 84 cases (10.3%). Univariate analysis showed that NLR was markedly higher in non‐survivors than in survivors (6.13 [2.48–14.0] vs. 3.10 [1.95–5.51]; *p* < 0.001; Table 2; Figure 3d). Similarly, patients who developed ICH had significantly elevated NLR compared with those without hemorrhage (5.08 [2.53–9.80] vs. 3.00 [1.90–5.45]; *p* < 0.001; Table 2; Figure 3c).

### 3.5 Predictive Accuracy of NLR for Functional Outcome at Discharge

ROC curve analysis demonstrated that NLR had moderate predictive performance for functional outcomes, with an area under the curve (AUC) of 0.679 (95% CI 0.641–0.717, *p* < 0.001), sensitivity of 61.5%, specificity of 68.2%, and an optimal cutoff value of 3.66. The predictive accuracy improved when NLR was combined with admission NIHSS scores, yielding an AUC of 0.849 (95% CI 0.822–0.875) (*p* < 0.001), with sensitivity of 76.8% and specificity of 79.0% (Figure 3).

**FIGURE 4:**
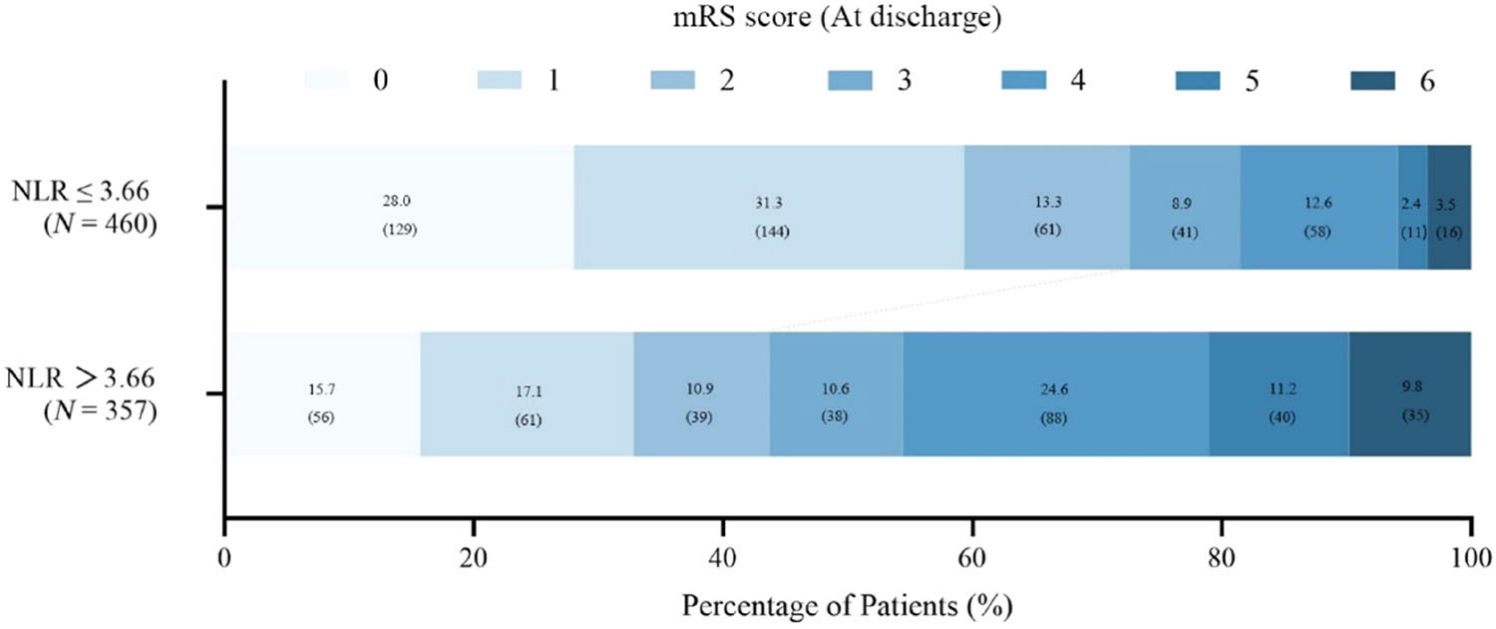
The mRS score at discharge for patients with higher NLR versus those with lower NLR. The cutoff value of the NLR for outcome is established through receiver operating characteristic (ROC) curves. mRS, modified Rankin Scale; NLR, neutrophil‐to‐lymphocyte ratio.

Using the optimal NLR cutoff value of 3.66, patients were divided into two groups. Individuals with an NLR > 3.66 were more likely to have unfavorable functional outcomes at discharge. The distribution of mRS scores between the two groups is presented in Figure 4.

## 4 Discussion

This study found that elevated NLR was independently associated with poor short‐term functional outcomes, in‐hospital mortality, and the occurrence of any ICH in AIS patients receiving reperfusion therapy. Furthermore, combining NLR with the admission NIHSS score improved its predictive accuracy for unfavorable functional outcomes. These results are consistent with previous research supporting NLR as a valuable prognostic biomarker in AIS patients treated with IVT or EVT.

Our study demonstrated that elevated NLR is associated with poorer outcomes in AIS patients, independent of the underlying cause of leukocyte elevation. Higher total leukocyte and neutrophil counts, as well as increased NLR, correlated with unfavorable outcomes following reperfusion therapies, including IVT (Malhotra et al. 2018) and EVT (Che et al. 2024). Elevated peripheral inflammation, reflected by higher NLR, appears to influence both immediate clinical response to reperfusion and longer‐term prognosis, supporting its role as an early prognostic marker. Previous studies have confirmed the predictive value of NLR in AIS (Quan et al. 2021), linking it to complications such as post‐stroke infections and HT (Sharma et al. 2021; Świtońska et al. 2020). Investigations by Duan et al. (2018) and Tokgoz et al. (2014) identified NLR as a risk factor for poor outcomes after EVT, while Lattanzi et al. (2017) reported an association between elevated NLR and early neurological deterioration. Compared with neutrophil count alone, NLR has been shown to be a more reliable predictor (Bi et al. 2021; Shi et al. 2018), especially in Asian populations (Xue et al. 2017). These findings, consistent with our results, underscore the significance of NLR as an inflammatory biomarker influencing clinical outcomes in AIS, even after successful recanalization. Moreover, most previous studies focused on patients treated with either IVT or EVT alone. Although some included both treatment groups, they analyzed them separately rather than as a unified reperfusion cohort. In contrast, our study assessed the overall reperfusion population, enabling a more comprehensive evaluation of the relationship between inflammatory response and functional outcomes. Additionally, we examined the impact of different reperfusion modalities—including IVT alone, EVT alone, and combined IVT plus EVT—on functional outcomes, which further confirmed the impact of NLR on the functional outcome of AIS patients undergoing reperfusion therapy.

Neutrophils and lymphocytes play key roles in the early inflammatory response following ischemic stroke (Neumann et al. 2015). Ischemic triggers the release of free radicals and inflammatory mediators, causing BBB disruption and tissue injury (Jickling et al. 2013; Rosell et al. 2008). Neutrophils are the first immune cells to infiltrate ischemic regions (Kim et al. 2016), initiating inflammation (Gong et al. 2021), whereas lymphocytes help regulate the post‐ischemic immune response and contribute to tissue repair. Their accumulation can exacerbate thrombosis, BBB leakage, endothelial swelling, and microvascular occlusion, contributing to the “no reflow” phenomenon (Schiphorst et al. 2020). The NLR serves as a systemic marker of this inflammatory response and has been linked to poor functional outcomes, particularly in large vessel occlusion patients undergoing EVT (Brooks et al. 2014).

HT is a common complication after reperfusion therapy. Studies suggest that higher NLR predicts a higher risk of sICH (Che et al. 2024; Ma et al. 2022) and post‐stroke complications, including infection (Brooks et al. 2014). NLR may also enhance clinical scoring systems, helping to identify patients at risk for adverse outcomes after EVT. Elevated NLR is linked to increased MMP‐9 release by neutrophils, which disrupts the BBB and contributes to sICH in patients post‐EVT (Jickling et al. 2013). Our study further supports this association, showing that NLR is strongly correlated with any ICH and in‐hospital mortality in AIS patients undergoing reperfusion therapy.

Beyond NLR, clinical factors such as age, baseline NIHSS score, and mRS score significantly influence outcomes after reperfusion. Advanced age is a recognized risk factor for futile recanalization, likely due to increased frailty and comorbidities (Bai et al. 2021). NIHSS provides an objective measure of stroke severity and strongly predicts post‐reperfusion outcomes (Hussein et al. 2018). These results highlight the multifactorial nature of stroke recovery and underscore the importance of integrating inflammatory markers with clinical parameters to guide prognosis and optimize treatment strategies.

## 5 Limitations

This study has several limitations. First, NLR was assessed only at admission, and the temporal changes in NLR during the course of the stroke and their effects on clinical outcomes remain unclear. Second, as a single‐center study, selection bias cannot be fully excluded, although our institution's status as a comprehensive stroke center may mitigate this concern. Third, the lack of a placebo‐controlled group limits the ability to definitively evaluate the impact of reperfusion therapy in patients with elevated NLR. Fourth, the retrospective design precludes establishing a causal relationship between NLR and outcomes, including whether interventions that reduce NLR would improve prognosis. Finally, the study primarily focused on short‐term outcomes at discharge, without examining 90‐day functional recovery. Future studies should investigate the predictive value of admission NLR for longer‐term outcomes, particularly at 90 days, in patients undergoing reperfusion therapy. Despite these limitations, this observational study provides valuable insights into the role of NLR in short‐term prognosis and safety after reperfusion, supporting its potential utility in guiding clinical decisions during the hyperacute phase of AIS.

## 6 Conclusions

Our findings indicate that NLR is an independent predictor of short‐term functional outcomes in AIS patients undergoing reperfusion therapy. Due to its accessibility, low cost, and ease of measurement, NLR may serve as a practical prognostic biomarker in clinical settings, aiding in the early identification of high‐risk patients and guiding timely interventions to mitigate adverse outcomes. Further studies should investigate its long‐term predictive value and consider stratified analyses based on infarct location, stroke subtype, and other relevant clinical variables.

## Author Contributions

**Lan‐ying He and Jing Gou**: conceptualization. **Lan‐ying He and Jun‐ying Li**: methodology. **Jun‐ying Li**: Formal analysis and investigation. **Jun‐ying Li**: Writing – original draft. **Jun‐ying Li, Jun Li, Zhong‐jiao Lu, Wen Zhou, Yan‐hui Li, Yong‐jiang Luo, Xue‐min Zhong, Jian Wang, Jing Gou, and Lan‐ying He**: Writing – review and editing. **Lan‐ying He**: funding acquisition. **Jun‐ying Li, Jun Li, Zhong‐jiao Lu, Wen Zhou, Yan‐hui Li, Yong‐jiang Luo, and Xue‐min Zhong**: resources. **Lan‐ying He, Jing Gou, and Jian Wang**: supervision.

## Funding

This work is supported by the Sichuan Medical and Health Care Promotion Institute (KY2022QN0355).

## Ethics Statement

This study received approved from the Ethics Committee of Chengdu Second People's Hospital.

## Conflicts of Interest

The authors declare no conflicts of interest.

## Supporting information




**Supporting Materials**: brb371122‐sup‐0001‐SuppMat.docx

## Data Availability

All data generated or analyzed during this study are included in this published article and its supplementary information files.
