# Supplementary material for: Neutrophil‐to‐Lymphocyte Ratio as an Independent Predictor of Adverse Short‐Term Functional Outcomes After Reperfusion Therapy in Acute Ischemic Stroke
Source: Brain Behav. 2025 Dec 10;15(12):e71122. doi: 10.1002/brb3.71122 (PMC12696041; doi:10.1002/brb3.71122)
Supplement: Supplementary file 1 — Supporting Materials: brb371122‐sup‐0001‐SuppMat.docx [file BRB3-15-e71122-s001.docx]

**Supplement**

Table S1 Compare the NLR value among the IVT, EVT and IVT+EVT groups

|  | IVT (N=556) | EVT (N=150) | IVT+EVT (N= 111) | *P* value |
| --- | --- | --- | --- | --- |
| NLR | 2.70 (1.77-4.40) | 4.92 (2.72-8.13) | 5.26 (2.48-9.11) | ＜0.001* |

Abbreviations: NLR, neutrophil-to-lymphocyte ratio; IVT, Intravenous thrombolysis; EVT, Endovascular treatment

Table S2 Compare the differences in NLR among different prognosis groups under IVT, EVT and IVT + EVT conditions respectively

|  | IVT | | | EVT | | | IVT+EVT | | |
| --- | --- | --- | --- | --- | --- | --- | --- | --- | --- |
|  | mRS 0-2 (N=411) | mRS 3-6（N=145） | *P* | mRS 0-2(N=42) | mRS 3-6(N=108) | *P* | mRS 0-2(N=37) | mRS 3-6(N=74) | *P* |
| NLR | 2.44(1.69-3.85 | 3.86(2.30-6.67) | ＜0.001* | 4.29(2.16-6.04 | 5.41(2.82-9.99) | 0.040* | 4.29(2.43-6.31) | 6.21(2.49-10.27) | 0.023* |

Abbreviations: NLR, neutrophil-to-lymphocyte ratio; IVT, Intravenous thrombolysis; EVT, Endovascular treatment; mRS, the modified Rankin scale
